# From action planning and plan enactment to fruit consumption: moderated mediation effects

**DOI:** 10.1186/s12889-017-4838-y

**Published:** 2017-10-23

**Authors:** Stefanie Kasten, Liesbeth van Osch, Sander Matthijs Eggers, Hein de Vries

**Affiliations:** 1 Department of Health Promotion, Faculty of Health, Medicine and Life Sciences, Maastricht University, PO Box 616, 6200 Maastricht, MD Netherlands; 2 CAPHRI-School for Public Health and Primary Care, Maastricht University, Maastricht, Netherlands

**Keywords:** Fruit consumption, Action planning, Plan enactment, Moderated mediation

## Abstract

**Objectives:**

Sufficient fruit consumption is beneficial for a healthy live. While many Dutch adults intent to eat the recommended amount of fruit, only 5–10% of the population actually adheres to the recommendation. One mechanism that can help to narrow this gap between intention and actual fruit consumption is action planning. However, action planning is only assumed to be effective if plans are enacted. This study assessed which action plans are made and enacted, and further aimed to investigate two main hypotheses: 1. the effect of action planning (at T1) on fruit consumption (at T2) is mediated by plan enactment (at T3); 2. positive intentions (2a), high self-efficacy (2b) and a strong habit to eat fruit (2c) enhance the mediation of plan enactment, whereas a strong habit to eat snacks (2d) hinders the mediation of plan enactment.

**Methods:**

This study was a self-reported longitudinal online survey study. A total of 428 participants filled in a survey, measuring demographic factors (e.g. gender, age, education level), several socio-cognitive constructs (i.e. attitudes, self-efficacy, habit, action planning, plan enactment), and fruit consumption, at three points in time (baseline, after 1 month, and after 3 months). Mediation and moderated mediation analyses were used to investigate the planning-plan enactment- fruit consumption relationship.

**Results:**

Up to 70% of the participants reported to have enacted their T1 action plans at T2. Action planning on fruit consumption was fully mediated by plan enactment (Hypothesis 1). All four proposed moderators (i.e. intention, self-efficacy, habit to consume fruit, and habit to consume snacks) significantly influenced the mediation (Hypotheses 2a-2d). Mediation of plan enactment was only present with high levels of intention, high levels of self-efficacy, strong habits to eat fruit, and weak habits to eat snacks.

**Conclusion:**

The study suggests the importance of plan enactment for fruit consumption. Furthermore, it emphasizes the necessity of facilitating factors. High levels of intention, self-efficacy and a strong habit to consume fruit clearly aid the enactment of action plans. This suggests that when these factors are moderately low, plan enactment may fail and thus an intervention may require first steps to foster these moderating factors.

## Background

A balanced diet based on low fat, vitamins, and fibers is beneficial for a healthy life and for maintaining body weight. To achieve such a diet the consumption of sufficient fruit and vegetables is essential. Fruit and vegetable products are the primary source of vitamines, minerals, and dietary fiber [1]. Studies have emphsized the importance of vegetable and fruit consumption with regard to the prevention of coronary heart disease, stroke, and hypertension. Additionally, fruit and vegetable consumption is inversely associated with overweight, type 2 diabetes mellitus, and even some forms of cancer [2]. To improve overall health the WHO (World Health Organisation) recomments a minimum of 400 g of fruit and vegetables per day for adults. However, amongst the Dutch adult population only 5% adheres to the guidelines for vegetable consumption and around 5–10% eats sufficient fruit [3–5]. While vegetables are mainly consumed during meals, fruit is mostly eaten between meals as a snack [5]. Aditionally to the critically low consumption of fruits and vegatables, the consumption of salt, often through the intake of salty snacks, is too high amongst the same population [4]. One option to reduce the intake of unhealthy snacks is to stimulate the consumption of fruit and thereby replace the high calorie dense snack with a healthy option [6, 7]. However, changing unhealthy dietary intake can be difficult, due to the fact that behaviour is highly influenced by several cognitive and environmental factors [8–10]. Cognitive determinants such as self-efficacy, intention, and habit have been studied to be important influential factors regarding healthy behaviour [11–13]. Although the impact of these factors on fruit consumption has been studied [14–16], little attention has been paid to their moderating effect on the process of behaviour change, and factors that have been shown to preceede and facilitate behaviour changes, such as intention and action planning.

Intention has long been regarded as the strongest and most proximal indicator for behavioural performance [17, 18]. Yet, studies show that people often fail to translate their good intentions into actions, a phenomenon widely known as the “intention-behaviour-gap” [19, 20]. To close this gap between the intention to eat healthy and actually consuming a healthier diet, a number of theorists have put forward planning models and strategies [19, 21, 22].

Several health behaviour change theories such as the Health Action Process Approach [23] (HAPA) and the I-Change Model [24] illustrate how planning of specific actions (‘action planning’) can help to bridge the intention-behaviour gap and therefore lead to more efficient behaviour change. Action planning refers to a process of creating a series of specific sub-actions to translate a person’s intention into actions aimed at goal achievement [21, 25–28]. However, this is only the case when the action plans are enacted [28–30]. Even though goal setting implies both planning and plan enactment, little is known about the separate contributions of plan enactment to behaviour change regarding health behaviour [24, 29, 30]. De Vries et al. [28] investigated the mediating effect of plan enactment in the relationship between action planning and smoking cessation and showed that the effect of action planning on behaviour was fully mediated by enactment. The first goal of this study is therefore to replicate previous findings of de Vries et al. [28] with regard to fruit consumption. Based on their findings, we hypothesize that plan enactment will mediate the effect of planning on fruit consumption (hypothesis 1).

A second goal of this study pertains to assessing whether the planning process is influenced by other factors (hypotheses 2a-2d). We will concentrate on four factors (i.e. intention, self-efficacy, and fruit and snack habit) that are hypothesized to moderate the effects of plan enactment. The first proposed moderator is intention. Studies found that high intentions strengthen the effect of planning on behaviour [31] and that people with high intentions enact more plans and thereby enhance behaviour change [28, 30]. Therefore, our second hypothesis is that the higher a person’s intention, the stronger the mediation effect of plan enactment (hypothesis 2a). The second proposed moderator of plan enactment is self-efficacy. Models like the HAPA and the I-Change Model state the importance of self-efficacy regarding behaviour change [23, 24]. However, self-efficacy has not only been influential with regard to behaviour. While Sniehotta et al. [32] found that self-efficacy acts as a mediator between intention and behaviour, other studies found that self-efficacy is one of the strongest predictors for planning [33, 34] and the enactment of plans [35]. Furthermore, high self-efficacy is assumed to result in more perseverance to attain certain goals [36]. Consequently, we assume more mediation of plan enactment under high levels of self-efficacy (hypothesis 2b). The third proposed moderator is habit. Habits may show an oppositional influence on behaviour change. A strong habit regarding healthy behaviour enhances health behaviour [37–39], whereas a strong habit with regard to unhealthy behaviour can be a barrier to healthy behaviour change [40, 41]. This indicates that whereas strong unhealthy nutrition habits can be identified as barriers for behaviour change, the formation of healthy habits has been found to be a strong indicator for increased consumption of fruit and vegetables [37]. We therefore hypothesize that a strong habit to consume fruit would strengthen the mediation by plan enactment (hypothesis 2c), and a strong habit to snack would inhibit the mediation effect (hypothesis 2d).

Within this study, we firstly explore and describe which specific action plans regarding fruit consumption are made at T1 and which of these action plans are enacted at T2. Subsequently, we will depict the relationship between planning, plan enactment and fruit consumption. Afterwards, we will investigate this relationship in the context of several social cognitive variables. We will test the hypothesis that plan enactment mediates the effect of action planning on fruit consumption (hypothesis 1), and the hypotheses that high intention, high self-efficacy and a strong habit to eat fruit will enhance plan enactment, whereas a strong habit to eat snacks may hinder the enactment of action plans with regard to fruit consumption (moderated mediation hypotheses 2a–d).

## Methods

### Participants and procedure

The sample of the conducted study consisted of Dutch adults (>18 years). All participants were registered members of an online survey panel (i.e. Flycatcher). Registration to the panel free of charge and can be done online. The panel contacts the members that are relevant for a specific study. In case of this study the panel contacted Dutch adults that were representative for the Dutch adult population with regard to gender, age, and educational level. There were no other inclusion criteria beside the minimum age of 18. A total of 806 participants were invited by e-mail, to participate in the online study. The email consisted of an explanation that confidentiality would be ensured, that the proposed study would contain three waves of measurements, and that the completion of all three questionnaires would be rewarded with a small incentive (approximately 3€). Additionally, the e-mail contained a link that led participants to the online questionnaire. Informed consent was obtained from all individual participants included in the study.

At baseline measurement (T1), 574 respondents (71.0%) completed the questionnaire. Four weeks later, at first follow-up (T2), 496 participants (86.4% of baseline) filled in the questionnaire. Finally, a total of 428 participants (74.6% of baseline) also completed the second follow-up (T3), eight weeks after baseline.

The subjects of this study were neither subjected to procedures, required to follow rules of behaviour, nor did the study involved scientific medical research. Therefore the study did not fall under the scope of the WMO (Medical Research Involving Human Subjects Act) and ethical approval was not required.

### Questionnaire

At baseline demographic variables, past fruit consumption behaviour, intention, self-efficacy, action plans and habit strength (regarding fruit and snack consumption) were measured. At T2 plan enactment was assessed, and at T3 the consumption of fruit was measured. All items were pre-tested by experts of the online panel. Items were tested on readability and comprehensiveness and adjusted accordingly.

#### Demographics (T1)

Participants were asked to indicate their gender, age and highest completed educational level. The educational level was classified as “low” (elementary education, medium general secondary education, preparatory vocational school, or lower vocational school), “medium” (higher general secondary education, preparatory academic education, or medium vocational school), and “high” (higher vocational school or university level).

#### Intention (T1)

Intention was measured by two items. The first item referred to the extent to which participants intended to consume two pieces of fruit daily. The second item added a time-reference, asking respondents to what extent they intended to consume fruit within the next month. Answering options for both items ranged from (1) “I definitely do not intend to” to (7) “I definitely intend to” (Cronbach’s α = 0.93). A mean score was calculated for further analyses.

#### Self-efficacy (T1)

Self-efficacy was assessed by four items to examine to what extent participants think they will be able to consume fruit in different situations [42], i.e. fruit consumption “during the week”, “during the weekend”, “when one is very busy”, and “during the winter months” (Cronbach’s α = 0.91). Answering options varied from (1) “I will certainly not be able to” to (7) “I will certainly be able to”. Throughout the analyses the mean score was used.

#### Habits (T1)

Habit strength was measured for the habit of eating fruit and the habit of eating snacks. Snacks were defined as high-caloric snacks, which were divided into 5 categories: 1. Fatty snacks (e.g. pizza, hamburgers), 2. Salty snacks (e.g. potato chips, nuts), 3. Sugary snacks (e.g. cookies, cake), 4. Candy bars, and 5. Savory snacks (e.g. sausage, dices of cheese). Habit strength was measured by a subset of six items from the Self-Report Habit Index [43]. Statements beginning with either fruit or snack “consumption is something” were followed by “I do frequently”, “I do automatically”, “that would require effort not to do”, “that belongs to my daily routine”, “I have no need to think about doing”, and “that’s typically “me”” (fruit consumption: Cronbach’s α = 0.95; snack consumption Cronbach’s α = 0.93). Answering options ranged from (1) “I totally disagree” to (7) “I totally agree”. A mean score was calculated and used throughout the analyses.

#### Action planning (T1)

Action planning was measured by five items that were derived from expert consulting and literature review [24, 44, 45]. Participants were asked to what extent they planned to perform a variety of sub-behaviours or actions, in order to reach higher consumption of fruit. Action plans included “buying fruit”, “eating fruit at a fixed time of the day”, “putting a fruit basket on the table”, “taking fruit along when leaving the house (e.g. to work)”, and “replacing unhealthy snacks with fruit” (Cronbach’s α = 0.77). Answering options varied from (1) “I am definitely not planning to” to (7) “I definitely plan to”. For the purpose of the frequency analysis, the variable was recoded into (0 = categories 1 to 4) plan was not made and (1 = categories 5 to 7) plan was made. For further analyses a mean score was calculated.

#### Plan enactment (T2)

Plan enactment was assessed by five items, measuring the extent to which respondents had carried out the five action plans in the last month (Cronbach’s α = 0.61). The question regarding buying more fruit could be answered on a scale from (1) “no, not more” to (4) “yes, a lot more”, whereas the other four questions had answering options ranging from (1) “no, (almost) never” to (4) “yes, (almost) always”. For the purpose of the frequencies variable was recoded into (0 = category 1) plan was not enacted and (1 = categories 2 to 4) plan was enacted. For further analyses a mean score was calculated.

#### Fruit consumption (T1, T2)

The fruit consumption measurement was based on a validated questionnaire [46] and included two items regarding a) the number of days per week the participant usually consumes fruit (0 to 7), and b) the average amount of fruit the participant consumes on each of these days. Multiplication of these two responses results in an accurate measure of the fruit consumption during a week.

### Statistical analysis

For the statistical analysis IBM SPSS Statistics 20 was used. To maximize available information given by the collected data, pairwise deletion was used to control for missing observations. Descriptive analyses were executed to describe the sample with regards to the background variables (age, sex, educational level, baseline fruit consumption, action plans, and plan enactment (T2)). Crosstabs and chi-square values were calculated to investigate the proportion of people who made action plans (T1) and who enacted these action plans (T2). In order to get insight into the associations between the variables Pearson’s correlation were calculated between outcome fruit consumption (T3), baseline fruit consumption (T1), action planning (T1), plan enactment (T2), intention (T1), self-efficacy (T1), habit of fruit consumption (T1), habit of snack consumption (T1), and each of the five action plans (T1) and plan enactments (T2) separately. With regard to hypothesis 1, mediation analysis was based on the INDIRECT macro recommended by Preacher and Hayes [41]. This technique measures the effect of an independent variable (X) on a dependent variable (Y) mediated by a mediator (M). First, path A indicates the effect of *X* on *M*. Path B, examines the effect of *M* on *Y*. Path C is the simple effect of the *X* on *Y* without controlling for *M*. Whereas C′ is the coefficient of path *X* ➔ *Y* after including *M* in the model. The indirect effect of *X* on *Y* through *M* is calculated by subtracting C′ from C (path AB = C - C′) [47]. Mediation was tested with plan enactment as a mediator of the relationship between action plans and the outcome behaviour. Subsequently, moderated mediation was tested using MODMED macro as suggested by Preacher et al. [48]. Moderated mediation was examined for intention (hypothesis 2a), self-efficacy (hypothesis 2b), habit strength of eating fruit (hypothesis 2c), and habit strength of eating snacks (hypothesis 2d). All analyses were adjusted for the background variables (i.e. gender, age, education and baseline fruit consumption).

## Results

### Description of sample

Background variables of participants are displayed in Table 1. In total, 574 participants filled out the online baseline questionnaire. The respondents’ mean age was 47.8 years (*SD* = 15.98) and 53.3% were women. Most participants had a medium level of education (42.5%) and the average fruit consumption per week at baseline was 8.3 pieces.

**Table 1:** Sample characteristics

	At Baseline T1 (*N* = 574)	At first follow-up T2 (*N* = 496)	At second follow-up T3 (*N* = 428)
Age in years: *M* (*SD*; Range)	47.83 (15.98; 19–87)		
Gender: female (%)	53.3		
Education (%):			
Low	26.3		
Medium	42.5		
High	31.2		
Fruit consumption (pieces per week): *M* (*SD*)	8.33 (6.71)		
Intention^a^: *M* (*SD*)	5.20 (1.41)		
Self-efficacy^a^: *M* (*SD*)	5.11 (1.25)		
Habit to eat fruit^a^: *M* (*SD*)	4.62 (1.70)		
Habit to eat snacks^a^: *M* (*SD*)	2.74 (1.51)		
Action planning^a^: *M* (*SD*)	4.52 (1.22)		
Plan enactment^b^: *M* (*SD*)		2.00 (0.68)	
Fruit consumption (pieces per week): *M* (*SD*)			8.21 (6.71)

^a^Measured on a scale ranging from 1 to 7

^b^Measured on a scale ranging from 1 to 4

### Action planning and plan enactment frequencies

Subsequently, frequency analysis was conducted to investigate which action plans have been made at T1 and which action plans were enacted at T2. Table 2 shows the percentages of participants that made action plans at T1 and enacted these action plans at T2. The plan to “take fruit along when leaving the house” was made most frequently (57.8%). However, the plans to “eat fruit at a fixed moment during the day” (69.4%) and to “take fruit along when leaving the house” (69.3%) were most often enacted at T2 when made at T1.

**Table 2:** Frequencies of action plans and their enactment

	Participants that made plan at T1 in % (*n*)	Participants that enacted their plan at T2 in % (*n*)
Action plan 1: “I am planning to buy more fruit”	54.4 (312)	50.0 (156)
Action plan 2: “I am planning to eat fruit at a fixed time of the day”	51.2 (294)	69.4 (204)
Action plan 3: “I am planning to put a fruit basket on the table”	45.8 (263)	65.8 (173)
Action plan 4: “I am planning to take fruit along when leaving the house (e.g. to work)”	57.8 (332)	69.3 (230)
Action plan 5: “I am planning to replace unhealthy snacks with fruit”	47.9 (275)	68.3 (174)

### Correlates of plan enactment

Correlations were calculated between outcome fruit consumption (T3), plan enactment (T2), baseline fruit consumption, action planning, intention, self-efficacy, habit of fruit consumption, habit of snack consumption, and each of the five action plans (T1) and plan enactment (T2) items separately (see Table 3) to investigate the associations between all variables used in the analyses.

**Table 3:** Correlations of plan enactment

Pearson correlation	1	2	3	4	5	6	7	8	9	10	11	12	13	14	15	16	17	18
1. Fruit consumption T3	1																	
2. Fruit consumption T1	.76^c^	1																
3. Intention T1	.34^c^	.40^c^	1															
4. Self-efficacy T1	.57^c^	.63^c^	.54^c^	1														
5. Habit snack T1	-.23^c^	-.21^c^	−.07	-.26^c^	1													
6. Habit fruit T1	.64^c^	.72^c^	.44^c^	.77^c^	-.26^c^	1												
7. Mean AP^a^ T1	.31^c^	.38^c^	.59^c^	.55^c^	−.06	.52^c^	1											
8. AP^a^ 1: Buying fruit T1	.10^c^	.09^c^	.52^c^	.30^c^	.02	.22^c^	.68^c^	1										
9. AP^a^ 2: Eating fruit at a fixed time of day T1	.37^c^	.44^c^	.46^c^	.50^c^	-.10^c^	.46^c^	.69^c^	.31^c^	1									
10. AP^a^ 3: Putting a fruit basket on the Table T1	.12^c^	.20^c^	.31^c^	.33^c^	−.08	.33^c^	.72^c^	.35^c^	.35^c^	1								
11. AP^a^ 4: Taking fruit along when leaving the house T1	.28^c^	.34^c^	.39^c^	.41^c^	−.03	.45^c^	.77^c^	.39^c^	.46^c^	.42^c^	1							
12. AP^a^ 5: Replacing unhealthy snacks with fruit T1	.27^c^	.29^c^	.48^c^	.46^c^	−.03	.40	.75^c^	.49^c^	.38^c^	.41^c^	.47^c^	1						
13. Mean PE^b^ T2	.46^c^	.50^c^	.40^c^	.48^c^	-.14^c^	.55^c^	.63^c^	.31^c^	.43^c^	.50^c^	.55^c^	.48^c^	1					
14. PE^b^ of AP^a^ 1 T2	.07	−.01	.18^c^	.04	.04	.02	.28^c^	.42^c^	.05	.18^c^	.16^c^	.24^c^	.42^c^	1				
15. PE^b^ of AP^a^ 2 T2	.53^c^	.59^c^	.29^c^	.50^c^	-.22^c^	.61	.38^c^	.07	.52^c^	.19^c^	.33^c^	.25^c^	.64^c^	−.04	1			
16. PE^b^ of AP^a^ 3 T2	.11^c^	.17^c^	.16^c^	.18^c^	−.07	.23^c^	.39^c^	.13^c^	.17^c^	.59^c^	.24^c^	.25^c^	.62^c^	.15^c^	.19^c^	1		
17. PE^b^ of AP^a^ 4 T2	.33^c^	.39^c^	.28^c^	.37^c^	−.04	.42^c^	.46^c^	.15^c^	.30^c^	.24^c^	.62^c^	.33^c^	.73^c^	.12^c^	.41^c^	.23^c^	1	
18. PE^b^ of AP^a^ 5 T2	.37^c^	.35^c^	.37^c^	.37^c^	-.11^c^	.38^c^	.47^c^	.29^c^	.27^c^	.30^c^	.37^c^	.48^c^	.72^c^	.30^c^	.32^c^	.36^c^	.52^c^	1

^a^ *AP* Action plan

^b^ *PE* Plan enactment

^c^sig. at *α* < .05

Our results show that planning to eat fruit at a fixed time, planning to take fruit along when leaving the house, and planning to replace unhealthy snacks by fruit displayed moderate correlations (*r* = 0.3–0.5) with the outcome behaviour, whereas plans to buy fruit and to place fruit on the table were only weakly correlated (*r* < 0.3). The strongest correlation between enactment of plans and the outcome variable was found between eating fruit at a fixed time, taking fruit along when leaving the house and replacing unhealthy snacks with fruit. Placing a fruit basket on a table was only weakly (*r* < 0.3) correlated, and the plan to buy fruit was not significantly correlated to the outcome. Furthermore, we explored the correlations of the moderating factors with later fruit consumption at T3. Baseline fruit consumption, habit of fruit consumption, self-efficacy, and plan enactment were strong (*r* > 0.5) to moderate (0.5 < *r* > 0.3) positively correlated with fruit consumption at T3. Action planning and intention on the other hand showed weaker correlations (*r* < 0.3). Additionally, a weak (*r* < −0.3) negative relationship between habit of snacking and fruit consumption at T3 was found.

### Plan enactment mediates action planning and fruit consumption (hypothesis 1)

**Fig. 1:**
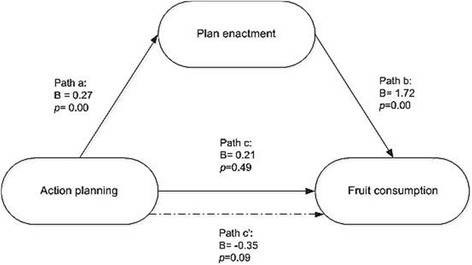
Mediation effects of plan enactment

To assess our first hypothesis, the mediation effect of plan enactment was investigated with the Preacher and Hayes technique (47). This analysis (see Fig. 1) yielded a significant effect of action planning on plan enactment (*B* = .27; *95% CI* = .23 — .31) and of plan enactment on fruit consumption (*B* = 1.72; *95% CI* = .96 — 2.48). Neither path C (*B* = .12; *95% CI* = −.23 — .47) nor path C′ (*B* = −.35; *95% CI* = −.76 — .06) indicated a significant effect of action planning on behaviour. However, results demonstrate that making action plans regarding fruit consumption at T1 led to a higher rate of plan enactment at T2, which subsequently affects the total fruit consumption measured at T3. This indicates that, although the sole act of planning did not have a direct influence on fruit consumption (path C), a significant indirect effect of action planning on fruit consumption via plan enactment was found (*B* = .47; *95% CI* = .26 — .71).

### Moderated mediation effects of intention, self-efficacy and habit strength (hypotheses 2a–d)

Subsequently, we examined the potential moderated mediation effects using the MODMED macro as suggested by Preacher et al. [48]. Table 4 shows the size of the indirect effect for each level of the moderator. The first proposed mediator was intention (hypothesis 2a). Results show a strong positive moderating effect for intention revealing that action plans made by participants at T1 were more often enacted when levels of intention were moderate or high (level ≥ 2; cut-off level: *β* = .49; *95% CI* = .15–.94). Only, when intention was at the lowest level (level = 1) there was no significant moderation found. With regard to self-efficacy, the second moderator (hypothesis 2b), we found similar effects. Participants’ action plans at T1 were more often enacted when a person scored high on self-efficacy (level ≥ 5; cut-off level: *β* = .39; *95% CI* = .19–.61). For low levels of self-efficacy, no significant mediation was present. With regard to habit, we hypothesized positive effects for habits to eat fruits (hypothesis 2c) and negative effects for an opposing habit, such as the habit to eat snacks (hypothesis 2d). For the habit to eat fruit a strong positive moderation was found when participants scored moderate or high (level ≥ 4; cut-off level: β = .23; *95% CI* = .04–.44), indicating that a strong habit to eat fruit can enhance the enactment of action plans. On the other hand, for the habit to consume snacks results show that only when the habit was low to moderate (level ≤ 4; cut-off level: β = .28; *95% CI* = .01–.56) enactment of action plans is enhanced.

**Table 4:** Regression models testing moderated mediation^a^

	Moderators							
	Intention		Self-efficacy		Habit Fruit		Habit Snacks	
	Coef	*95% CI*	Coef	*95% CI*	Coef	*95% CI*	Coef	*95% CI*
Conditional indirect effects^b^								
Level = 1	.46	.05–1.02	−.05	−.56–.14	−.01	−.28–.16	.70***	.37–1.08
Level = 2	.49*	.15–.94	−.01	−.48–.20	.04	−.23–.23	.54***	.31–.81
Level = 3	.51**	.21–.88	.07	−.29–.32	.12	−.12–.31	.40***	.18–.63
Level = 4	.53***	.29–.79	.21	−.07–.41	.23*	. 04–.44	.28*	.01–.56
Level = 5	.53***	.30–.78	.39***	.19–.61	.38***	.18–.62	.18	−.14–.54
Level = 6	.53***	.23–.87	.61***	.35–.93	.57***	.29–.90	.09	−.31–.51
Level = 7	.51*	.07–1.00	.89***	.43–1.43	.79***	.37–1.29	.02	−.44–.50

^a^:Probed values based on the Johnson-Neyman technique, with bootstrapped confidence intervals (5000 samples)

^b^: All analysis were corrected for baseline fruit consumption, sex, age and educational level

*p < .05, ** *p* < .01, *** *p* < .001

## Discussion

The importance of planning for health behaviour change has been investigated and proven across various health behaviours [23, 49, 50]. However, little research has been done concerning the significance of plan enactment and which factors may be associated with increased enactment.

Concerning the explorative analyses, our results reveal that between 50 to 70% of the participants reported to have enacted their T1 action plans at T2. Although the plan to “take fruit along when leaving the house” was made most frequently, the plan to “eat fruit at a fixed moment during the day” was most often enacted at T2. Furthermore, these two action plans also showed the strongest correlations with fruit consumption at T3. Even though the action plans to buy fruit and put fruit in a visible place have also been made and enacted often, both the planning and the enactment show only weak correlations with actual fruit consumption, indicating that these action plans are not strongly predictive of fruit consumption. A reason could be that people tend to buy fruit and may not consume it or only partly [51].

Our first hypothesis stated that the effect of action plans on fruit consumption would be mediated by plan enactment. Our findings showed that the effect of action planning on fruit consumption was fully mediated by plan enactment. This replicated findings of an earlier study showing that plan enactment mediated the effect of planning on smoking cessation behaviour [28]. Our results emphasize the importance of correct enactment of action plans when it comes to health behaviour change. This suggests that making specific action plans and executing them correctly can influence fruit consumption over the long term. Earlier research, as well as this study, indicates that guiding individuals to make plans is helpful but often insufficient for behaviour change when plans are not correctly or fully enacted [28, 52]. The results indicate that action planning is only a part of the behaviour change and that without the enactment of the plans behaviour change is difficult to reach [13].

With regard to hypotheses 2 a-d stating that the mediation of plan enactment would be moderated by different factors, our findings identified significant moderation of the mediation effect through all four proposed moderators (i.e. intention, self-efficacy and fruit and snack habit). Our moderated mediation analyses revealed that the higher participants’ intention was to consume fruit at baseline the stronger was the mediating effect of plan enactment (hypothesis 2a). This indicates that high intentions can facilitate the enactment of action plans. There was no moderating effect found on the b-paths suggesting that when a plan has been enacted the effect on fruit consumption would not depend on the level of intention. These findings support our second hypothesis and are in line with findings of earlier studies about smoking cessation and sunscreen use [28].

Next, our results regarding the third hypothesis showed that the mediation effect of enactment between action planning and fruit consumption was stronger when a person’s level of self-efficacy was also high (hypothesis 2b). These moderated mediation result support earlier findings showing that high levels of self-efficacy can act as an effective facilitator for behaviour change with regard to fruit and vegetable consumption [53], as well as an influential factor regarding goal achievement [12, 32, 34]. The results also replicate earlier findings by Lippke et al. [35], who indicated that when a person lacks self-efficacy, planning may be ineffective. However, no significant moderation was found concerning the b-path between enactment of action plans and fruit consumption indicating that independent from the level of self-efficacy the enactment of action plans is related to fruit consumption. This contradicts findings by Barz et al. [54]. Their study showed that the level of self-efficacy moderated the b-path between enactment of preparatory behaviours and the outcome behaviour, indicating that in the case of physical activity the use of preparatory actions would be more helpful for people with low self-efficacy. In other words, while the level of self-efficacy did not matter with regard to the enactment of a plan, high levels of self-efficacy were essential when it came to the translation of the enacted action plans into broader physical activity (e.g. making the plan to buy sport shoes led to buying shoes disregardful of the self-efficacy level, but buying sport shoes lead only to more physical activity when the level of self-efficacy was high). While our study did find support for moderation on the effect of action planning on enactment, the study by Barzet al. [54] found that self-efficacy moderates the effect of enactment on behaviour. This disparity could be explained by the differences in necessity of preparatory actions for each behaviour, and by the difficulty of these actions [54–56]. This indicates that more research is needed with regard to different health behaviours. Furthermore, the contrast in findings emphasizes the need for more research with regard to plan enactment and moderators across different health behaviours.

Third, we expected and found that the stronger a participant’s habit to eat fruit the more likely it was that action plans were enacted (hypothesis 2c). Furthermore, a reversed moderating effect was found for the habit to eat snacks, meaning that only when the habit to eat snacks is weak, a significant mediation effect is found (hypothesis 2d). The results imply that action planning is less effective when opposing habits exist. Our results are in line with earlier studies [57–60] and show that action planning is helpful when increasing already habitual behaviour [40, 61, 62]. However, action planning alone might not be sufficient to change behaviour when a person has a strong antagonizing habit [58, 59]. There was no moderating effect found for the b-path, indicating that as soon as a plan has been enacted habit has no significant effect on the plan enactment-behaviour relationship.

These results propose that it is important to understand under which conditions plans are enacted (e.g. high intention, high self-efficacy, and a strong habit to eat fruit). Interventions need to ensure that these fostering conditions are fulfilled to assure behaviour change. This suggests that when these factors are moderately low, plan enactment may fail and thus an intervention may require first steps to booster these moderating factors. Research therefore needs to focus on possibilities to monitor and follow-up on the enactment of plans during an intervention more closely. Additionaly, the identification of factors such as self-efficacy and intention facilitating or hindering plan enactment need further attention in order to make action planning interventions more effective.

Some limitations to our study should be mentioned. First, within our study no differences in specificity or quality of the action plans made were assessed. Previous studies showed that the more specific a plan is, the greater its predictive value [27, 63]. This leads to the assumption that more specific and higher quality action plans would also lead to more enactment, a hypothesis that needs to be substantiated in future studies. Second, in our study we made no differentiation of plan enactment per plan, meaning that we only investigated the overall effect of making action plans and enacting them. Looking at each plan separately could give us further insight in which action plans are more often enacted and are more likely to be influenced by third factors such as intention or habit.

Furthermore, we did not differentiate between conditional (e.g. if situation *Y* occurs I will do *X*) and unconditional plans (e.g. I will do *X*, independent of any situation *Y*). Sniehotta [64] argues that the distinction is necessary to understand the exact effect of planning on behaviour. Some studies have demonstrated the effectiveness of conditional plans with regard to behaviour change [65, 66]. Yet, other research has shown that making more specific conditional plans (e.g. If I leave my house at 8:00 am, then I will take an apple) might not be suitable for unconditional behaviour change, because when situation Y does not occur the action will not be triggered and thereby the behaviour is unaffected [64]. This leads to the assumption that unconditional plans may have a greater effect on behaviour change because their effect is not bound to specific contexts. However, in order to test the process of planning and the importance of the working mechanism of the planning-enactment-behaviour relationship further research is needed to determine whether it will also be important to discriminate between conditional and unconditional plans.

Another limitation that needs to be mentioned is that fruit consumption was measured in pieces of fruit that were consumed per day. While this method is validated [46] one can argue that nowadays a lot of fruit is consumed in forms of juices, sauces or even stewed. However research shows that most health effects can be found when fruits are consumed solid and fresh [67]. An additional limitation is the relative high dropout rate. While online surveys are relatively easy to conduct and often cost-effective the anonymity and lack of direct contact between participants and researcher can lead to lower response rates [68]. Other reasons for high dropout can be the questionnaire lengths or level of interest of the participants in the study topic. However, even though the dropout rate was relatively high, the sample was in terms of gender, age and educational level still representative for our population. Finally, within this study no control group was included. The inclusion would have led to a clearer picture about the effectiveness of the plans and their enactment.

The present study contributes to current research of fruit consumption, in particular with regard to the importance of plan enactment. Evidence is provided, that action planning alone is not automatically beneficial to behaviour change and that whether or not a person enacts a plan is dependent on levels of intention, self-efficacy and habit. Furthermore, our results support findings with regard to mediation and moderation in other health behaviour domains such as action control with regard to physical activity [34], condom use [69], physical activity [54] and smoking cessation [28].

## Conclusion

The results of this study indicate the important role of plan enactment in fruit consumption, but also show that enactment of action plans is often suboptimal and depends on factors such as intention, self-efficacy, and habit. Furthermore, the results show that when action plans are not enacted behaviour change is less likely to occur [29]. The study suggests that interventions should not only target the construction of action plans, but also should monitor and support the enactment of these action plans, by for example sending reminders or boosters. Furthermore, the moderated mediation effects suggest that planning interventions should consider other factors such as intention, self-efficacy, and antagonistic habit to ensure that action planning actually leads to behaviour change. To further substantiate these findings and to strengthen the foundation of plan enactment further research, both for verification and generalization would be necessary.

## Abbreviations

AP: Action plans
HAPA: Health Action Process Approach
PE: Plan enactment
